# The Risk of Exposure to Ticks and Tick-Borne Pathogens in a Spa Town in Northern Poland

**DOI:** 10.3390/pathogens11050542

**Published:** 2022-05-04

**Authors:** Katarzyna Kubiak, Małgorzata Dmitryjuk, Janina Dziekońska-Rynko, Patryk Siejwa, Ewa Dzika

**Affiliations:** 1 Department of Medical Biology, Collegium Medicum, School of Public Health, University of Warmia and Mazury in Olsztyn, Zolnierska 14c, 10-561 Olsztyn, Poland; e.dzika@uwm.edu.pl; 2 Department of Biochemistry, Faculty of Biology and Biotechnology, University of Warmia and Mazury in Olsztyn, Oczapowskiego 1A, 10-719 Olsztyn, Poland; m.dmit@uwm.edu.pl; 3 Department of Zoology, Faculty of Biology and Biotechnology, University of Warmia and Mazury in Olsztyn, Oczapowskiego 1A, 10-719 Olsztyn, Poland; jdr@uwm.edu.pl (J.D.-R.); patryk.siejwa12@gmail.com (P.S.); 4 Students’ Parasitology “Vermis” Science Club, Department of Medical Biology, Collegium Medicum, School of Public Health, University of Warmia and Mazury in Olsztyn, Zolnierska 14c, 10-561 Olsztyn, Poland

**Keywords:** *Ixodes ricinus*, *Dermacentor reticulatus*, *Borrelia burgdorferi* sensu lato, *Anaplasma phagocytophilum*, *Rickettsia* spp., *Babesia* spp., Poland

## Abstract

The aim of this study was to determine the potential risk of human exposure to tick-borne infection in a recreation areas in a spa town located in northern Poland. Questing *Ixodes ricinus* and *Dermacentor reticulatus* ticks were collected in the spring of 2018. Tick-borne microorganisms were detected by PCR. Species were identified based on RFLP and the sequencing of DNA. In total, 38.3% of the ticks (34.6% of *I. ricinus* and 48.6% of *D. reticulatus*) were infected. The prevalence was 14.9% for *Borrelia* spp., 10.6% for *Babesia* spp. and 17.7% for *Rickettsia* spp. No *Anaplasma phagocytophilum* was detected. Spirochaetes *B. afzelii*, *B. garinii* and *B. burgdorferi* s.s. were detected only in *I. ricinus* ticks (20.2%). The differences in the infection rates of *Babesia* spp. between *I. ricinus* (7.7%) and *D. reticulatus* (18.9%) were not significant. DNA of *B. canis* and *B. venatorum* were identified in both tick species. *B. microti* were detected in *D. reticulatus* ticks. The prevalence of *Rickettsia* spp. was significantly higher in *D. reticulatus* (37.8%) than that in *I. ricinus* (10.6%). *R. raoultii* was identified only in *D. reticulatus* and *R. helvetica* in *I. ricinus*. Co-infections of at least two pathogens were recognized in 13% of positive ticks.

## 1. Introduction

In the northern hemisphere, the most commonly diagnosed vector-borne diseases in humans and animals are infections transmitted by ticks, mainly by the *Ixodes* and *Dermacentor* genera. In northern Europe, including Poland, the most widely distributed tick species are *I. ricinus* and *D. reticulatus* [1,2].

*I. ricinus* is involved in the transmission of the tick-borne encephalitis virus—the causative agent of tick-borne encephalitis (TBE), a potentially fatal neurological infection [3]—and bacteria from the *Borrelia burgdorferi* sensu lato (s.l.) complex, which cause the multisystemic disease Lyme borreliosis (LB) [4]. *I. ricinus* is also known as a vector of bacteria belonging to the order Rickettsiales, *Anaplasma phagocytophilum*—the agent of human granulocytic anaplasmosis [5,6]—and *Rickettsia* spp., including *R. helvetica* and *R. monacensis*, whose pathogenicity in humans is controversial; however, they may cause a mild illness similar to a spotted-fever-like disease [7]. *I. ricinus* can also transmit parasitic protozoa of the genus *Babesia* spp.—*B. divergens*, *B. venatorum* and *B. microti*, responsible for human babesiosis [8]. In turn, *D. reticulatus* is a common vector of the protozoa *B. canis*, the causative organism of canine babesiosis, and also bacteria of the genera *Rickettsia* spp. and *Anaplasma* spp., as well as tick-borne encephalitis virus [9,10]. The significance of *D. reticulatus* in the transmission of the spirochaete *B. burgdorferi* s.l. is still unclear [10].

In Poland, among the diseases transmitted by ticks, only cases of LB and TBE are officially registered. In 2016–2019, LB was diagnosed in over 20,000 and TBE in ~250 patients every year [11]. Such a large number of registered cases of tick-borne diseases (TBDs) indicate a high risk of also acquiring other infections, including human granulocytic anaplasmosis, tick-borne rickettsioses and human babesiosis [5,12,13].

Exposure to tick-borne infections is not only associated with being in natural biotopes that provide ticks a living environment with appropriate microclimatic conditions and access to hosts necessary for the completion of the tick life cycle, but also in urban green spaces, such as parks, gardens and recreational areas [14,15,16,17]. Due to the lack of data about the prevalence of tick-borne pathogens in urban areas in north-eastern Poland (the Warmia–Mazury province), where the incident rate of LB is almost twice as high as that in the rest of Poland [11], the aim of this study was to determine the potential risk of human exposure to tick-borne infection with *Borrelia* spirochaetes, *A. phagocytophilum*, *Rickettsia* spp. and protozoa from the *Babesia* genus in a recreational area of a spa town in northern Poland (north-eastern part of the Warmia and Mazury regions).

## 2. Results

### 2.1. Identified Tick Species

A total of 141 questing ticks were collected during the springtime activity of ticks in 2018 at three sampling sites in a spa town in the north-eastern part of the province of Warmia–Mazury, Poland. Among them, 104 (73.6%) were identified as *I. ricinus* (51 females, 39 males and 14 nymphs) and 37 were determined as *D. reticulatus* (24 females and 13 males). The mean density ranged from 2.4 to 6 ticks per 100 m^2^.

### 2.2. Prevalence and Diversity of Pathogens

The DNA of at least one of the four microorganisms was revealed in 38.3% (54/141) of the examined tick genomic DNA isolates. Overall, the prevalence of the examined pathogens was 14.9% (n = 21) for *Borrelia* spp., 10.6% (n = 15) for *Babesia* spp. and 17.7% (n = 25) for *Rickettsia* spp. The DNA samples of all ticks tested were negative for *A. phagocytophilum*. Among the positive PCR samples, 34.6% (36/104) were from *I. ricinus* and 48.6% (18/37) from *D. reticulatus*.

#### 2.2.1. *Borrelia* spp.

DNA of spirochaetes from the *B. burgdorferi* s.l. complex was detected only in *I. ricinus* ticks. Among them, 20.2% (21/104) were *Borrelia*-positive. (Table 1). The highest percentage of *Borrelia* spp. was recorded among *I. ricinus* males (23.1%, 9/39), followed by females (21.6%, 11/51) and nymphs (7.1%, 1/14). The differences between developmental stages were not statistically significant (Table 1). The species typing performed on the basis of the RFLP patterns revealed the presence of DNA of *B. afzelii*, *B. garinii* and *B. burgdorferi* s.s. As a monoinfection, the DNA of *B. afzelii* was detected in 52.4% (11/21) of ticks and *B. garinii* in 42.1% (8/21). In one female and one male, *B. afzelii* was in co-infection with *B. garinii* and *B. burgdorferi* s.s., respectively (Table 2).

Sequences of amplicons of the *flaB* gene of *B. afzelii* (n = 4) were identical (GenBank: OM927743) and showed 100% nucleotide identity with those of the BO23 and K78 strains, which are pathogenic to humans (GenBank: CP018262, CP009058). These sequences were also 100% identical to *B. afzelii* sequences derived from *I. ricinus* (GenBank: MW595226) and *D. reticulatus* (GenBank: MW595227) ticks feeding on deer from the Warmia–Mazury province and from questing *I. ricinus* ticks from northern (GenBank: KX6461950) and south-northern (GenBank: KF836511) Poland. Sequences of *B. garinii* (n = 7) were also monomorphic (GenBank: OM927742) and showed 100% nucleotide identity to the sequences of *B. garinii* obtained from naturally infected questing *I. ricinus* ticks from a different part of Poland and the Czech Republic (GenBank: MK604254, HM 345905, JN828685).

#### 2.2.2. *Babesia* spp.

*Babesia* spp. DNA was identified in 7.7% (8/104) of *I. ricinus* and in 18.9% (7/37) of *D. reticulatus* ticks. The differences in the infection rates between tick species were not statistically significant (Table 1). In *I. ricinus*, the DNA of *Babesia* spp. was detected only in females (7.8%, 4/51) and nymphs (28.6%, 4/14) (χ^2^ = 11.8, *p* < 0.05). In *D. reticulatus*, the infection rate did not differ statistically between females (12.5%, 3/24) and males (30.8%, 4/13).

Ten *Babesia*-positive PCR products were sequenced. Among sequences from *I. ricinus,* the DNA of *B. canis* (n = 6) and *B. venatorum* (n = 1) was identified (Table 2). *B. canis* (n = 1), *B. venatorum* (n = 1) and *B. microti* (n = 1) were detected in *D. reticulatus*.

All partial sequences of the *18S rRNA* gene identified as *B.* canis were identical. Manually checking the *B. canis* chromograms revealed a double pick of nucleotides (GA/AG) at the position corresponding to the 609–610 nucleotides of the complete *18S rRNA* gene (GenBank: AY072926). This combination of nucleotides is characteristic of genotype II of *B. canis*. All obtained sequences showed 100% similarity to *B. canis* detected in the blood of naturally infected dogs from Poland (GenBank: EU622793) and Croatia (GenBank: AY072926), as well as in questing *I. ricinus* ticks (GenBank: KR003829) and ticks removed from human skin (GenBank: MW791420) in Poland. A similar sequence was also derived from questing *D. reticulatus* in Poland (GenBank: KT272401).

The nucleotide sequences of *B. venatorum* identified in nymphs of *I. ricinus* and males of *D. reticulatus* were identical and showed 100% similarity to *B. venatorum* isolated from human patients in Italy (GenBank: KJ663730) and Austria (GenBank: AY046575), as well as from blood of the roe deer in the Czech Republic (GenBank: MG344777). This sequence variant was also reported earlier in questing *I. ricinus* ticks (GenBank: KC007116) and those removed from human skin (GenBank: MW791418), respectively, in Germany and in Poland.

The single *B. microti* sequence detected in a *D. reticulatus* male was identical to genetic variants classified as non-pathogenic *B. microti* Munich-type (GenBank: AB071177, AY789075).

#### 2.2.3. *Rickettsia* spp.

*Rickettsia* spp. was detected in 17.7% (25/141) of analysed ticks. There were significant differences in the infection rate (χ^2^ = 13.9, *p* < 0.001) between *I. ricinus* (11/104, 10.6%) and *D. reticulatus* (14/37, 37.8%) (Table 1). In *I. ricinus*, the DNA of *Rickettsia* spp. was the most frequently confirmed in males (15.4%, 6/39), followed by females (7.8%, 4/51) and nymphs (7.1%, 1/14). In *D. reticulatus*, the highest percentage of infected ticks was recorded among females (45.8%, 11/24), rather than in males (23.1%, 3/13). Differences in the infection rates between developmental stages in both tick species were not statistically significant (Table 1).

To identify the *Rickettsia* species, 15 amplicons of the *gltA* gene were sequenced. Comparison with the data registered in GenBank revealed the presence of *R. helvetica* (n = 7) and *R. raoultii* (n = 8). *R. helvetica* was identified only in *I. ricinus* and *R. raoultii* in *D. reticulatus* ticks (Table 2).

All obtained *R. helvetica* sequences were identical and showed 100% nucleotide similarity to sequences of the *R. helvetica* strain C9P9 (GenBank: U59723) from ticks. Identical sequences of the *R. helvetica gltA* gene were also obtained from questing *I. ricinus* in central Poland (GenBank: MH018961–78) and from *I. ricinus* feeding on wild ungulates in north-eastern Poland (MW595234–595237).

Eight sequences of the *gltA* gene of *R. raoultii* were monomorphic and fully similar to sequences of the *R. raoultii* strain IM16 isolated from a human sample in China (GenBank: CP019435), from questing *D. reticulatus* in Poland (GenBank: KT277489) and from *Haemaphysalis longicornis* (GenBank: MN450401) and *Dermacentor silvarum* (GenBank: MH046861) ticks from China.

#### 2.2.4. *Borrelia* spp., *Babesia* spp. and *Rickettsia* spp. Co-Infections

Co-infections of at least two pathogens were recognized in 13% (7/54) of positive samples (Table 3). In *I. ricinus* ticks, in equal proportions, double (*B. afzelii*/*B. canis* and *B. venetorum/R. helvetica*) and triple (*B. afzelii/B. garinii/R. helvetica* and *B. afzelii/B. burgdorferi* s.s./*R. helvetica*) co-infections were identified. In *D. reticulatus*, only co-infections of *Babesia* spp. and *Rickettsia* spp. were detected. *R. rauoltii* was confirmed in two cases of co-infections.

## 3. Discussion

The assessment of the potential risk posed by the transmission of TBDs in a specific area is a complex process that requires knowledge of tick presence and tick-borne pathogen infection rates [14,18]. The present study focused on molecular evidence of pathogens transmitted by ticks in a recreational urban area in a region of north-eastern Poland, where the incident rate of LB is almost twice as high as that in the rest of Poland [11]. Despite the limitations of our research resulting from the number of collected *I. ricinus* and *D. reticulatus* ticks from green areas in the city of Gołdap in northern Poland and the collection of ticks only in the springtime of their activity during one year, we confirmed earlier results that urban green spaces are an environment favourable to ticks [15,16,19,20,21]. The low tick population density recorded in the study area (2.4 to 6 ticks per 100 m^2^) is typical for urban areas, where the mean density is nearly 2–3 times lower compared to that in natural ecotypes [16,22,23]. However, it provides further evidence that members of the public within sites may be at risk from tick bites and acquiring TBDs.

In our study, every third tick (38.3%) was infected with at least one of examined microorganisms, although the infection rates differed between *I. ricinus* and *D. reticulatus*. From an epidemiological point of view, the *I. ricinus* tick has greater importance than *D. reticulatus*. *I. ricinus* dominated among ticks removed from human skin in Europe [24,25,26] and it is considered to be a main vector of Lyme borreliosis [27]. In our study, spirochaetes from the *B. burgdorferi* complex were detected only in *I. ricinus*. Although the specific DNA of that bacteria has been detected in *D. reticulatus* in other regions of Poland [10,28,29,30] their role as a vector was not confirmed. Among the examined *I. ricinus* ticks, 20% of them were infected with *Borrelia*, with the predominance of pathogenic *B. afzelii* (52.4%) and *B. garinii* (42.1%). These two species are considered to be the most abundant in Europe [4]. Such a high level of infection rate in *I. ricinus* has already been recorded in green urban spaces in north-eastern Poland. In Olsztyn, the capital of the Warmia and Mazury region, almost 36% of ticks feeding on dogs and 27% of questing ticks were infected with *Borrelia* spirochaetes [15,29]. The level of *Borrelia* spp. infection in *I. ricinus* in north-eastern Poland is higher than that recorded in other regions of the country (0.25–12.4%) [31,32], and is also higher than that in the green areas of other large European cities (4–18%) [16,20,33,34]. Comparable infection rates were calculated only in the *I. ricinus* population in large cities in Hanover, Germany (22.7%) [35], and in Hamburg (34.1%) [36]. The meta-analysis conducted on the results of studies from 2010–2016 in Central European countries revealed that 19.3% of *I. ricinus* were *Borrelia*-positive [4]. In our study, we noticed that the infection rate of adult ticks (~22%) was three times higher than that of nymphs (7.1%), which confirmed the general trend [4,32].

Bacteria from the *Rickettsia* genus were the second-most-frequent microorganism detected in the examined ticks from the recreational area in the city of Gołdap. In total, almost 18% of them were confirmed to have *Rickettsia* DNA. The level of infection in *D. reticulatus* was over three times higher (37.8%) than that in *I. ricinus* (10.6%). The results of the DNA sequencing of the *gltA* gene suggested that *D. reticulatus* was infected mainly with *R. raoultii* and *I. ricinus* with *R. helvetica*. The rate of infection *in D. reticulatus* was slightly lower than that revealed in ticks feeding on wild ungulates (49%) in north-eastern Poland [28] and also in the population of questing *D. reticulatus* of other regions of Poland (40.7–44%) [10,30,37,38,39]. In European countries, the percentage of infected *D. reticulatus* with *Rickettsia* spp. may reach even 70–80% of the population of questing *D. reticulatus* [40,41]. European populations of *I. ricinus* are most often infected with *R. helvetica* and are much less infected with *R. monacensis*, *R. raoultii*, *R. sibirica* and *R. slovaca* [17,42]. The prevalence of *R. helvetica* in *I. ricinus* varies depending on the study location and ranges from 0.5% to about 20% [17]. The infection level of *I. ricinus* in the present study (10.6%) was within this range and did not differ from the infection level established for *I. ricinus* in other regions of Poland (3–27.5%) [38,43,44,45]. Interestingly, *Rickettsia* spp. are significantly more frequently detected in *I. ricinus* in urban sites than in natural sites of Poland [43,45]. It is worth noting that both *Rickettsia* species identified in our study are pathogenic to humans and may be responsible for infections of varying severity with non-specific symptoms that often coexist with skin lesions. These symptoms are often unnoticed or misdiagnosed [7,43].

In Europe, including Poland, *I. ricinus* ticks are also a known vector of *A. phagocytophilum* bacteria. Although cases of human granulocytic anaplasmosis [5,46,47] and the presence of its DNA in *D. reticulatus* have been reported [29,43], we have not confirmed the occurrence of this pathogen in the population of ticks in the recreational areas of Gołdap. This may be related to the general level of their infection, which may vary locally from 0.91 to 14.40% in Poland and from 0.4 to 20% and more in other European countries [43,48].

We did not find differences in the prevalence of *Babesia* spp. between *I. ricinus* and *D. reticulatus*. In Europe, *I. ricinus* ticks are involved mainly in the transmission cycles of *B. divergens*, *B. microti* and *B. venatorum* (formerly *Babesia* sp. EU1) [49]. From this group of *Babesia* species in *I. ricinus* from urban green areas in northern Poland, we noted the occurrence of the human pathogen *B. venatorum*. Analysis of the 18S rDNA sequence showed similarity with *B. venatorum* isolated from patients in Italy and Austria [50]. It is worth noting that this piroplasm appears able to infect immunocompetent humans and can present with severe symptoms [50]. *B. venatorum* has already been detected in questing *I. ricinus* ticks from rural and urban areas in northern (0.9%) [51], eastern (1.2%) [52] and west-central [53] Poland, and also in other Baltic countries [54,55,56]. Interestingly, most of sequenced *Babesia* DNA in examined *I. ricinus* ticks was recognized as *B. canis*, which is vectored by *D. reticulatus*. The first European evidence for the presence of *B. canis* in questing *I. ricinus* was reported in urban and rural recreational areas in northern (0.2–1%) [51,57] and also in west-central (5%) Poland [53]. DNA of those piroplasms was also revealed in 0.2–1.4% of *I. ricinus* from Slovakia and the Czech Republic [58,59]. Liberska et al. [53] suggested that *I. ricinus* may locally play a role as an alternative vector for *B. canis* to dogs in areas where this tick species occurs much more often than *D. reticulatus*. However, it cannot be ignored that the *B. canis* DNA in *I. ricinus* may have originated from blood meals taken from infected dogs. Dwużnik et al. [60] showed that “contamination” with the blood of the host is the cause of the detection of *B. microti* DNA in *D. reticulatus* ticks. Identified by us and other authors [10,30,52], *B. microti*-positive questing *D. reticulatus* are probably the result of feeding on voles (*Microtus* spp.), the main reservoir hosts of *B. microti* and the major source of tick contamination with *B. microti* DNA.

From a medical point of view, co-infections by ticks with several species of pathogenic microorganisms are of significant importance. In the green recreational areas of the city of Gołdap, we revealed co-infections of all detected pathogens in various combinations in 13% of positive ticks. Tick co-infection and the co-transmission of pathogens might have important relevance to public health. Indeed, co-infection in humans might enhance disease severity and may also have significant consequences in terms of tick-borne disease treatment and diagnosis [47,61]. Moreover, even when the bites are not associated with the transmission of pathogens, they represent discomfort that can last longer than it should if they cause severe complications, e.g., anaphylactic shock and secondary infections that can further complicate the healing of the affected tissue [62].

## 4. Materials and Methods

### 4.1. Study Area and Tick Collection

**Figure 1:**
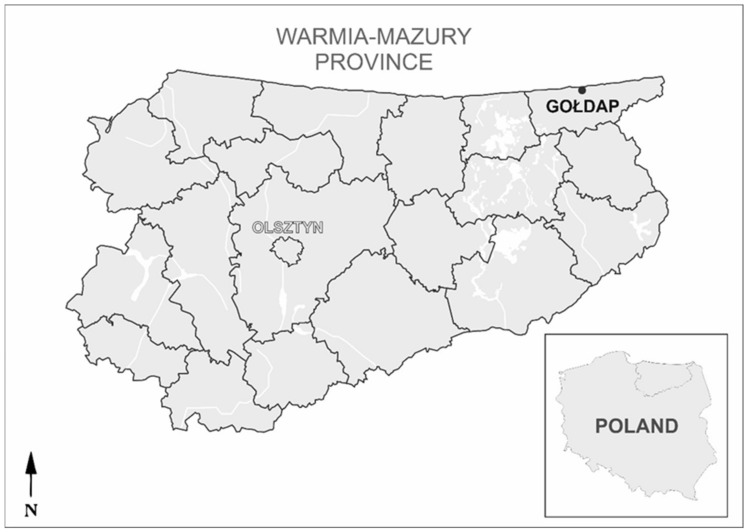
The location of the tick collection area (Gołdap) in the Warmia and Mazury region, northern Poland (Olsztyn, the capital of Warmia and Mazury). The map was designed in CorelDRAWX5 based on Google Maps (https://www.google.pl/maps; accessed on 20 June 2021).

This study was conducted in the city of Gołdap (54°18′22″ N, 22°18′13″ E) (Figure 1), a spa town located in the border area between Poland and Russia in the north-eastern part of the province of Warmia–Mazury. The city of Gołdap (~14,000 residents) is the only spa town in the region, specializing in the treatment of diseases of the musculoskeletal system, upper and lower respiratory tract and psychosomatic diseases. As a mud and climatic health resort with the best air quality in Poland, it is visited by approximately 70,000 patients and tourists each year.

The tick collection sites (surfaces of 300–350 m^2^) were situated in selected recreational areas visited by residents, tourists and patients of health resorts. The questing ticks were collected between April and May (the springtime activity of ticks) of 2018 during the daytime between 9 a.m. and 4 p.m. by one person for at least 30 min using the standard flagging method. In the laboratory, the collected ticks were identified by species, sex and developmental stage using taxonomic keys [63,64] and preserved in 70% ethanol for further molecular investigation.

### 4.2. DNA Extraction

Extraction of genomic DNA from ticks was carried out using a Sherlock AX universal kit (A&A Biotechnology, Poland) according to the manufacturer’s instructions. Prior to DNA extraction, the ticks were air-dried for several minutes and then individually cut and crushed with a sterile scalpel. DNA was isolated from individual specimens of ticks. The extracted DNA was eluted in 40 μL of TE buffer and stored at −20 °C for further analysis.

### 4.3. Pathogenic DNA Detection 

The presence of *Borrelia* spirochaetes in tick genomic DNA isolates was confirmed by screening with the use of conventional PCR and primers SL1/SL2 - amplified fragment of the outer surface protein A (*ospA*) gene [65] (Table 4) under the following reaction conditions: initial denaturation at 95 °C for 5 min, 40 cycles of denaturation at 95 °C for 30 s, primer annealing at 65 °C in 1 min and elongation a 72 °C for 1 min. Subsequently, *ospA*-positive samples were analysed by PCR with the use of a *Borrelia* spp. flagellin (*flaB*) gene with primers BFL1/BFL2 [66] (Table 4) according to the protocol described by Michalski et al. [29].

To detect *A. phagocytophilum* DNA, the EHR521 and EHR747 primers [67] (Table 4) targeting a fragment of the *16S rRNA* gene were used. Conventional PCR was conducted according to the protocol described by Michalski et al. [29].

The presence of *Babesia* spp. in tick genomic DNA samples was confirmed by using the sets of primers BJ1/BN2 specific to the *18S rRNA* gene (Table 4). The primers and thermal profiles used in this study are described in Casati et al. [68].

*Rickettsia* spp. DNA was detected by PCR under the thermal conditions described by Michalski et al. [28] using the CS409 and Rp1258 primers [69] (Table 4) amplifying a fragment of the *gltA* gene.

All amplifications were performed in a total volume of 25 µL of PCR mixture containing 12.5 µL of 2 × PCR Master Mix Plus (0.1 U/µL of Taq polymerase supplied in a PCR buffer, 4 mM of MgCl2 and 0.5 mM of each dNTPs) (A&A Biotechnology, Gdynia, Poland), 0.5 µL of each primer (10 µM), 4–5 µL of template DNA and an appropriate amount of sterile nuclease-free water. DNA of *B. canis* isolated from the blood of an infected dog and *B. afzelii*, *A. phagocytophilum* and *Rickettsia* spp. obtained from an infected *I. ricinus* tick (confirmed by sequencing in an earlier study) was used as a positive control. PCR amplicons were visualised on 1.5% agarose gels stained with Midori Green Stain (Nippon Genetics Europe, Düren, Germany) using GelDocXR (Bio-Rad, Hercules, CA, USA).

### 4.4. Identification of Pathogen Species

To confirm the species of the detected microorganisms, selected PCR products were purified using the CleanUp purification kit (A&A Biotechnology, Gdynia, Poland) according to the manufacturer’s protocol and sequenced bi-directionally (Macrogen Europe, Amsterdam, The Netherlands) with forward and reverse primers. The obtained nucleotide sequences were edited in BioEdit v. 7.2 software (https://bioedit.software.informer.com, accessed on 18 February 2022) and compared with data registered in the GenBank database (http://www.ncbi.nih.gov/Genbank/index.html, accessed on 18 February 2022) using the BLAST-NCBI program (http://www.ncbi.nlm.nih.gov/BLAST/, accessed on 18 February 2022). Consensus sequences were deposited in the GenBank database and registered under the accession numbers OM927742-43 for the *flaB* gene of *Borrelia* spp., OM913494, OM913540, OM913544 for the *18S rRNA* gene of *Babesia* spp. and OM927744-45 for the *gltA* gene of *Rickettsia* spp.

Additionally, to identify the *Borrelia* species, the restriction fragment length polymorphism (RFLP) method was used [66]. All positive *flaB* PCR products (422 bp) were digested with the *TasI* endonuclease (Fast Digest Tsp 509I, Thermo Fisher Scientific, Waltham, MA, USA) to obtain the restriction patterns of the three genospecies from *B. burgdorferi* s.l. complex: *B. garinii*, *B. afzelii* and *B. burgdorferi* s.s., and coinfections. Digestion reactions were carried out according to the manufacturer’s instructions. Restriction fragments were separated by electrophoresis on 3% agarose gel and stained with Midori Green dye (Nippon Genetics Europe GmbH, Düren, Germany).

### 4.5. Statistical Analysis

For each collection site, the density of ticks was estimated by determining the number of ticks per 100 m^2^ for each flagging event. A chi-square test or Fisher’s exact test (when the expected frequency was <5 in at least one of the cells of the contingency table) and 95% confidence intervals (95% CI) were used to compare differences in the prevalence of detected microorganisms between species and the developmental stages of questing ticks. The analysis was conducted using the software package SPSS version 27.0 for Windows (SPSS Inc., Chicago, IL, USA). In all analyses, *p*-values below 0.05 were considered statistically significant.

## 5. Conclusions

In our study, we confirmed that urban green areas in northern Poland are a favourable habitat for both common tick species in Poland—*I. ricinus* and *D. reticulatus*. We revealed the presence of pathogenic microorganisms in both species of ticks and in their developmental stages, when attacking people. *I. ricinus* carries a potentially high risk of human exposure to infection with *Borrelia* spirochaetes and a lower risk with *R. helvetica*. *D. reticulatus;* on the other hand, is potentially the main source of infection with pathogenic *R. raoultii* bacteria. For the course of tick-borne diseases acquired by humans in this area, the possibility of co-infection with several pathogens is not without significance.

## Figures and Tables

**Table 1:** Prevalence of *Borrelia* spp., *Babesia* spp., *Rickettsia* spp. and *Anaplasma phagocytophilum* in questing ticks in a recreational area of a spa town in northern Poland.

Tick Species and Developmental Stage		No. of Tested Ticks	No. of Infected Ticks n/% (95% CI)						
			*Borrelia*		*Babesia*		*Rickettsia*		*Anaplasma*
*I. ricinus*	F	51	11/21.6	*p* = 0.419 *	4/7.8	*p* = 0.003 *	4/7.8	*p* = 0.456 *	0/0
			(11.3–35.3)		(2.2–18.9)		(2.2–18.9)		
	M	39	9/23.1		0/0		6/15.4		0/0
			(11.1–39.3)				(5.9–30.5)		
	N	14	1/7.1		4/28.6		1/7.1		0/0
			(0.2–33.9)		(8.4–58.1)		(0.2–33.9)		
	Subtotal	104	21/20.2		8/7.7		11/10.6		0/0
			(13.0–29.2)		(3.4–14.6)		(5.4–18.1)		
*D. reticulatus*	F	24	0/0		3/12.5	*p* = 0.213 *	11/45.8	*p* = 0.288 *	0/0
					(2.7–32.4)		(25.6–67.2)		
	M	13	0/0		4/30.8		3/23.1		0/0
					(9.1–61.4)		(5.0–53.8)		
	Subtotal	37	0/0		7/18.9		14/37.8		0/0
					(8.0–35.2)		(22.5–55.2)		
			*p* = 0.003 *		*p* = 0.057 *		*p* < 0.001 *		

* chi^2^ test or Fisher’s exact test, *p* < 0.05; abbreviation: F—females, M—males, N—nymphs.

**Table 2:** Tick-borne microorganism species distribution in questing ticks in a recreational area of a spa town in northern Poland.

Pathogen Species		*I. ricinus* (n/%)	*D. reticulatus* (n/%)
*Borrelia* *	*B. afzelii*	11/52.4	0/0
	*B. garinii*	8/42.1	0/0
	*B. afzelii*/*B. garinii*	1/4.8	0/0
	*B. afzelii*/*B. burgdorferi* s.s.	1/4.8	0/0
*Babesia* **	*B. venatorum*	1/14.3	1/33.3
	*B. canis*	6/85.7	1/33.3
	*B. microti*	0/0	1/33.3
*Rickettsia* ***	*R. helvetica*	7/100	0/0
	*R. raoultii*	0/0	8/100

* Confirmed by the PCR-RFLP method (n = 21) and DNA sequencing (n = 11), ** confirmed by DNA sequencing (n = 10), *** confirmed by DNA sequencing (n = 16).

**Table 3:** Co-infections of questing ticks from recreational areas of a spa town in northern Poland.

Tick Species	Tick Stage	No. of Specimens	Pathogen Species
*I. ricinus*	F	1	*Borrelia afzelii*/*Babesia canis*
	F	1	*Borrelia afzelii*/*Borrelia garinii*/*Rickettsia helvatica*
	M	1	*Borrelia afzelii*/*Borrelia burgdorferi* s.s./*Rickettsia helvatica*
	N	1	*Babesia venetorum*/*Rickettsia helvatica*
*D. reliculatus*	F	2	*Babesia* spp./*Rickettsia raoultii*
	M	1	*Babesia* spp./*Rickettsia* spp.

Abbreviations: F—females, M—males, N—nymphs.

**Table 4:** Primer sets used for PCR amplification.

Pathogen	Primer Name	Primer sequence 5′–3′	Product Size [bp]	Gene Target	Reference
*Borrelia* spp.	SL1	AATAGGTCTAATAATAGCCTTAATAGC	307	*ospA* ^1^	[65]
	SL2	CTAGTGTTTTGCCATCTTCTTTGAAAA			
	BFL1	GCTCAATATAACCAAATGCACATG	422	*flab* ^2^	[66]
	BFL2	CAAGTCTATTTTGGAAAGCACCTAA			
*A. phagocytophilum*	EHR521	TGTAGGCGGTTCGGTAAGTTAAAG	247	16S rRNA	[67]
	EHR747	GCACTCATCGTTTACAGCGTG			
*Babesia* spp.	BJ1	GTCTTGTAATTGGAATGATGG	~490	18S rRNA	[68]
	BN2	TAGTTTATGGTTAGGACTACG			
*Rickettsia* spp.	CS409	CCTATGGCTATTATGCTTGC	769	*gltA* ^3^	[69]
	Rp1258	ATTGCAAAAAGTACAGTGAACA			

^1^ *ospA*—outer surface protein A gene, ^2^ *flaB*—flagellin gene, ^3^ *gltA*—citrate synthase gene.

## Data Availability

Not applicable.
